# Pretreatment lymphocytopenia is an adverse prognostic biomarker in advanced‐stage ovarian cancer

**DOI:** 10.1002/cam4.1956

**Published:** 2019-01-16

**Authors:** Yong Jae Lee, Young Shin Chung, Jung‐Yun Lee, Eun Ji Nam, Sang Wun Kim, Sunghoon Kim, Young Tae Kim

**Affiliations:** ^1^ Department of Obstetrics and Gynecology Institute of Women's Life Medical Science Yonsei University College of Medicine Seoul Korea

**Keywords:** immune suppression, lymphocytopenia, ovarian cancer, prognosis

## Abstract

The aim of this study was to investigate the prognostic significance of lymphocytopenia in advanced‐stage ovarian cancer. We retrospectively reviewed 506 patients with advanced‐stage ovarian cancer at Yonsei Cancer Hospital. This study included two cohorts of patients: a neoadjuvant chemotherapy (NAC) group (N* *=* *247) and a primary debulking surgery (PDS) group (N* *=* *259). The absolute lymphocyte count was recorded before treatment. A receiver operating characteristic (ROC) curve analysis was used to determine the cutoff for defining lymphocytopenia in the NAC cohort and followed by multivariate analysis. Subsequently, lymphocytopenia was assessed in the PDS cohort by multivariate analysis. A further analysis was performed to evaluate the absolute lymphocyte count as a continuous variable. An absolute lymphocyte count of 1.49 × 109/L was determined as the cutoff for the ROC curve analysis in the NAC cohort, and the multivariate analysis revealed that lymphocytopenia was an independent prognostic factor for poor progression‐free survival (PFS) [hazard ratio (HR), 1.50; 95% confidence interval (CI), 1.07‐2.11] and overall survival (OS) (HR, 2.02; 95% CI, 1.21‐3.40). In the PDS cohort, the multivariate analysis showed that lymphocytopenia was an independent prognostic factor for poor PFS (HR, 1.73; 95% CI, 1.20‐2.49) and OS (HR, 1.87; 95% CI, 1.27‐2.75). The absolute lymphocyte count was a significant factor when analyzed as a continuous variable in both the NAC and PDS cohorts. Pretreatment lymphocytopenia is an independent adverse prognostic factor in patients with advanced‐stage ovarian cancer.

## 1 INTRODUCTION

Epithelial ovarian cancer is a highly lethal gynecologic malignancy, and its incidence and mortality rates in Korea are increasing.^1^ The current standard of treatment includes primary debulking surgery (PDS) plus adjuvant platinum‐based chemotherapy for advanced ovarian cancer.^2^ Recently, several Phase 3 clinical trials have investigated whether rates of survival, postoperative morbidity, and mortality after receiving neoadjuvant chemotherapy (NAC) followed by interval debulking surgery (IDS) are not inferior to those achieved with PDS in women with Stages III‐IV ovarian cancer.^3^, ^4^ Therefore, NAC, followed by IDS, is an alternative approach to treat advanced‐stage ovarian cancer when complete resection cannot be achieved.

Clinical stage, histological subtypes and grade, residual tumor, the presence of ascites, performance status, and cancer antigen 125 (CA‐125) levels are all known prognostic factors in ovarian cancer.^5^ In addition to these established prognostic factors, interest has been growing in evaluating the systemic inflammatory and immune responses to cancer cells represented by the levels of neutrophils, lymphocytes, platelets, and acute‐phase proteins. A previous study showed that the systemic inflammation initiated by cancer induces apoptosis and margination of white blood cells leading to an increase in the number of neutrophils and a decrease in the number of lymphocytes.^6^ The absolute lymphocyte count is the number of lymphocytes circulating in peripheral blood, and deviations in this count can be used to evaluate the host's immune response during illness.^7^ Preoperative lymphocytopenia has been suggested recently as a new independent prognostic factor for several solid tumors, such as renal,^8^ bladder,^9^ and colorectal cancer.^10^ However, the prognostic significance of lymphocytopenia in ovarian cancer has not been evaluated. Lymphocytopenia may represent a type of cancer‐induced immune suppression that may limit the use of chemotherapy in cancer treatment.^11^

To investigate the prognostic significance of pretreatment lymphocytopenia, we chose a cohort of advanced‐stage ovarian cancer patients treated with PDS followed by adjuvant chemotherapy and a separate cohort of advanced‐stage ovarian cancer treated with NAC followed by IDS.

## 2 MATERIALS AND METHODS

The study was retrospective and included two cohorts of patients. There were 537 advanced‐stage ovarian cancer patients treated with NAC followed by IDS or PDS from 2006 to 2017 in our institution. The patients with autoimmune disease or patients taking medication that could affect the immune function were excluded in both cohorts. We excluded 31 patients in this study. Of 31 patients, 26 patients had autoimmune disease including thyroid disease, type 1 diabetes mellitus, and systemic sclerosis. Five patients were taking a medication with hepatitis. Finally, a total of 506 patients were included in the study.

The blood samples were collected in ethylenediaminetetraacetic acid (EDTA) tubes and immediately sent for analysis. Hematologic parameters were counted by XN‐9000 (Sysmex, Kobe, Japan) hematology analyzers. Absolute lymphocyte counts were calculated by multiplying the values of white blood cells by lymphocyte percentage.

### 2.1 NAC cohort

This cohort comprised 247 patients with pathologically confirmed epithelial ovarian cancer who had received at least one cycle of NAC at Yonsei Cancer Hospital between 2006 and 2017. Before starting NAC, all patients were histologically or cytologically confirmed to have Stage III or Stage IV epithelial ovarian cancer according to the criteria of the International Federation of Gynecology and Obstetrics (FIGO). The diagnosis was reached via either laparoscopic or image biopsy samples or by using fine‐needle aspiration of a tumor site or ascites/effusion. The histological diagnoses were based on World Health Organization criteria, and all microscopic slides were reviewed by two experienced gynecologic pathologists. NAC was performed if at least one of the following three criteria were met: (a) pulmonary and/or hepatic parenchymal metastases were observed on imaging studies before surgery, (b) the patient was medically inoperable, and/or (c) optimal cytoreduction was not achievable because of a high tumor burden (Fagotti score ≥ 8) observed by diagnostic laparoscopy.^12^ According to our institutional policy, IDS was preferred after three cycles of NAC. All patients received taxane and platinum combination chemotherapy.

During the period between the initial diagnosis and the initiation of NAC, all patients underwent at least two blood tests that included blood counts. This analysis used the lowest absolute lymphocyte count obtained during these tests.

### 2.2 PDS cohort

A total of 259 patients with pathology‐confirmed FIGO Stage III or IV ovarian cancer who underwent PDS at Yonsei Cancer Hospital between 2006 and 2014 were included in this cohort.

All underwent cytoreductive surgery and received postoperative adjuvant chemotherapy. During the period between the initial diagnosis and the PDS, all patients underwent at least two blood tests that included blood counts. This analysis used the lowest absolute lymphocyte count obtained during these tests.

### 2.3 Surgical procedures for PDS/IDS

Standard surgical procedures included the sampling of free fluid or peritoneal washings for cytology; a thorough inspection of the abdomen and pelvis, including the upper abdominal viscera, diaphragm, and retroperitoneal spaces; and hysterectomy, bilateral oophorectomy and omentectomy, pelvic/para‐aortic lymph node dissection, and appendectomy. If necessary, the surgery also included bowel resection, diaphragm or other peritoneal surface stripping, splenectomy, partial hepatectomy, partial gastrectomy, or partial cystectomy with or without ureteroneocystostomy, cholecystectomy, and/or distal pancreatectomy.^13^

### 2.4 Analysis

All patients’ medical records were reviewed retrospectively to collect clinical and laboratory data. The endpoints included progression‐free survival (PFS) and overall survival (OS), which were evaluated by RECIST (version 1.1). PFS was defined as the interval between the dates of diagnosis and the first recurrence. OS was defined as the interval between the dates of diagnosis and death or the last follow‐up. Recurrence was defined as the date of the appearance of radiologically detected disease during a follow‐up examination.

Descriptive data are reported as the median (range) or frequency (percentage). Categorical variables were compared with the chi‐square or Mann‐Whitney *U* test and continuous variables with Student's *t* test. Survival analyses used the Kaplan‐Meier method, and the data were compared using a log‐rank test. A receiver operating characteristic (ROC) curve was generated and assessed to find the best cutoff point within an absolute lymphocyte count to use to predict survival based on detection of lymphocytopenia in the NAC cohort. Based on the area under the ROC curve (AUC), the optimal cutoff (Youden index) was selected to maximize the sum of the sensitivity and specificity in the NAC cohort. A Cox regression analysis was used to evaluate the association of the prognostic factors with survival, expressed as hazard ratios (HR) and 95% confidence intervals (CI).

We adjusted the following variables for multivariate analysis: age (NAC cohort: >55 or ≤55, PDS cohort: >58 or ≤58), American Society of Anesthesiologists (ASA) score (1‐2 or 3‐4), absolute neutrophil count (≥1.49 × 10^9^/L or <1.49 × 10^9^/L), hemoglobin (≥12.0 g/dL or <12.0 g/dL), absolute lymphocyte count (≤7.5 × 10^9^/L or >7.5 × 10^9^/L), CA‐125 level (NAC cohort: ≤1715.3 U/mL or >1715.3 U/mL, PDS cohort: ≤1791.7 U/mL or >1791.7 U/mL), FIGO stage (III or IV), histologic subtypes (HGSC or non‐HGSC), residual disease (none or any residual), chemotherapy regimen (paclitaxel‐carboplatin or other), and cycles of total chemotherapy. Continuous variables such as age, absolute neutrophil count, hemoglobin, and CA‐125 were categorized according to median values. A second analysis evaluated the impact of the absolute lymphocyte count, absolute neutrophil count and hemoglobin, age, CA‐125 level, and cycles of total chemotherapy as continuous variables. For all analyses, the level of statistical significance was set to *P *<* *0.05. The statistical analyses were performed with the SPSS statistical software (version 21.0; IBM Corp., Armonk, NY).

## 3 RESULTS

### 3.1 NAC cohort

The ROC curve analysis determined an absolute lymphocyte count of 1.49 × 10^9^/L as the optimal cutoff (Figure S1). The area under the curve, sensitivity, and specificity were 0.67, 76%, and 49%, respectively. The clinical characteristics of the two groups are shown in Table 1. A total of 247 patients were included in this cohort; of these, 150 (60.7%) had lymphocytopenia and 97 (39.3%) did not. The two groups had significantly different median hemoglobin levels (*P *=* *0.015). We performed an analysis of the trend of absolute lymphocyte counts from 2006 to 2017 in NAC cohort and 2006 to 2014 in PDS cohort. There was no significant trend of absolute lymphocyte counts according to year (Table S1).

**Table 1:** Baseline characteristics of patients treated with NAC (N = 247)

Characteristics	Absolute lymphocyte count ≤1.49 × 10^9^/L (n = 150)	Absolute lymphocyte count >1.49 × 10^9^/L (n = 97)	*P*‐value
Age, median (range), y	57 (31‐80)	60 (27‐79)	0.175
ASA score, n (%)			
1	28 (18.7%)	13 (13.4%)	0.500
2	90 (60.0%)	57 (58.8%)	
3	30 (20.0%)	26 (26.8%)	
4	2 (1.3%)	1 (1.0%)	
CA‐125 level, median (range), U/mL	1781.5 (44.3‐20685.7)	1509.0 (66.7‐17911.3)	0.161
Hemoglobin level, median (range), g/L	12.1^ ^g/L (8.3‐16.3)	12.5^ ^g/L (9.4‐15.7)	0.015
Absolute lymphocyte count, median (range), cells/L	1.09 × 10^9^/L (0.31‐1.47)	1.85 × 10^9^/L (1.50‐3.10)	<0.001
Absolute neutrophil count, median (range), cells/L	5.40 × 10^9^/L (0.66‐14.02)	5.15 × 10^9^/L (1.49‐36.52)	0.316
FIGO stage, n (%)			
III	72 (48.0%)	40 (41.2%)	0.360
IV	78 (52.0%)	57 (58.8%)	
Histologic type, n (%)			
HGSC	137 (91.3%)	89 (91.8%)	0.986
Endometrioid	2 (1.3%)	1 (1.0%)	
Mucinous	2 (1.3%)	2 (2.1%)	
Others	9 (6.0%)	5 (5.2%)	
Grading			
1	5 (3.3%)	3 (3.1%)	0.121
2	23 (15.3%)	11 (11.3%)	
3	100 (66.7%)	77 (79.4%)	
Not available	22 (14.7%)	6 (6.2%)	
Residual disease, n (%)			
No	53 (35.3%)	45 (46.4%)	0.212
Any residual	82 (54.7%)	45 (46.4%)	
Not available	15 (10.0%)	7 (7.2%)	
Chemotherapy regimen, n (%)			
Paclitaxel + carboplatin	113 (75.3%)	81 (83.5%)	0.286
Docetaxel + carboplatin	34 (22.7%)	14 (14.4%)	
Paclitaxel + carboplatin + bevacizumab	3 (2.0%)	2 (2.1%)	
Cycles of total chemotherapy, median (range)	8 (4‐12)	8 (3‐12)	0.225

ASA, American Society of Anesthesiologists; CA‐125, cancer antigen 125; FIGO, International Federation of Gynecology and Obstetrics; HGSC, high‐grade serous carcinoma; NAC, neoadjuvant chemotherapy.

**Figure 1:**
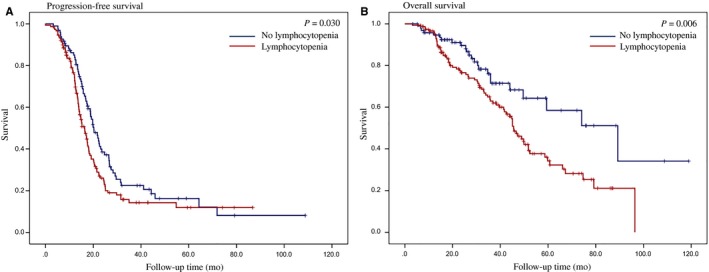
Kaplan‐Meier curves of progression‐free (A) survival and overall survival (B) stratified by lymphocytopenia in patients treated with NAC. NAC, neoadjuvant chemotherapy

The median follow‐up duration was 31.2 months (range, 1.6‐119.0 months). At the time of analysis, 99 patients (40.1%) had died, and 174 (70.4%) had recurrences. The Kaplan‐Meier curves for OS and PFS are shown in Figure 1. Patients in the lymphocytopenia group had poorer PFS (*P *=* *0.030) and OS (*P *=* *0.006) than those in the nonlymphocytopenia group. The median PFS was 16.3 months (range 2.1‐86.8 months) in the lymphocytopenia group and 20.2 months (range 3.2‐108.8 months) in the nonlymphocytopenia group. The median OS was 45.6 months (range 3.2‐96.4 months) in the lymphocytopenia group and 89.2 months (range 3.4‐119.0 months) in the nonlymphocytopenia group.

The results of the multivariate analysis of PFS and OS in patients treated with NAC are shown in Table 2. The multivariate analysis showed that pretreatment lymphocytopenia was an independent prognostic factor associated with a higher risk of progression (HR, 1.50; 95% CI, 1.07‐2.11) and inferior OS (HR, 2.02; 95% CI, 1.21‐3.40). Under multivariate analysis in a continuous variable model, the absolute lymphocyte count retained significance as a prognostic factor in recurrence (HR, 0.63; 95% CI, 0.46‐0.88) and OS (HR, 0.31; 95% CI, 0.17‐0.56). The results of the univariate/multivariate analyses for all variables are in Tables S2 and S3.

**Table 2:** Multivariate analyses for progression‐free and overall survival using a Cox proportional hazards model with categorical variables and continuous variables in patients treated with NAC

Variables	PFS	OS
	HR (95% CI)	HR (95% CI)
Absolute lymphocyte count (Categorical variables^a^)		
≥1.49 × 109/L	1	1
<1.49 × 10^9^/L	1.50 (1.07‐2.11)	2.02 (1.21‐3.40)
Absolute lymphocyte count (Continuous variables^b^)	0.63 (0.46‐0.88)	0.31 (0.17‐0.56)

ASA, American Society of Anesthesiologists; CI, confidence interval; FIGO, International Federation of Gynecology and Obstetrics; HR, hazard ratio; NAC, neoadjuvant chemotherapy; PFS, progression‐free survival; OS, overall survival.

a The multivariate analysis was adjusted for categorical variables (age, ASA score, hemoglobin, absolute neutrophil count, CA‐125 level, FIGO stage, histology, residual disease after IDS, chemotherapy regimen, cycles of total chemotherapy).

b The multivariate analysis was adjusted for continuous variables (age, hemoglobin, absolute neutrophil count, CA‐125 level, cycles of total chemotherapy) and categorical variables (ASA score, FIGO stage, histology, residual disease after IDS, chemotherapy regimen).

### 3.2 PDS cohort

The baseline characteristics of the cohort of patients treated with PDS are shown in Table 3. This cohort had 259 patients; of these, 172 (66.4%) had lymphocytopenia. The ASA score, median hemoglobin level, and median absolute neutrophil count showed significant differences between the lymphocytopenia and the nonlymphocytopenia groups.

**Table 3:** Baseline characteristics of patients treated with PDS (N = 259)

Characteristics	Absolute lymphocyte count ≤1.49 × 10^9^/L (n = 172)	Absolute lymphocyte count >1.49 × 10^9^/L (n = 87)	*P*‐value
Age, median (range), y	55 (22‐83)	54 (27‐83)	0.609
ASA score, n (%)			
1	71 (41.3%)	59 (67.8%)	0.001
2	82 (47.7%)	24 (27.6%)	
3	18 (10.5%)	4 (4.6%)	
Not available	1 (0.6)	0 (0%)	
CA‐125 level, median (range), U/mL	930.1 (21.5‐30008.8)	624.8 (12.1‐12000.0)	0.090
Hemoglobin level, median (range), g/L	11.7^ ^g/L (6.6‐16.7)	12.5^ ^g/L (9.9‐14.0)	<0.001
Absolute lymphocyte count, median (range), cells/L	1.05 × 10^9^/L (0.23‐1.48)	1.84 × 10^9^/L (1.49‐3.05)	<0.001
Absolute neutrophil count, median (range), cells/L	6.48 × 10^9^/L (2.16‐21.51)	5.07 × 10^9^/L (2.04‐14.21)	<0.001
FIGO stage, n (%)			
III	122 (70.9%)	65 (74.7%)	0.521
IV	50 (29.1%)	22 (25.3%)	
Histologic type, n (%)			
HGSC	135 (78.5%)	71 (81.6%)	0.663
Endometrioid	11 (6.4%)	6 (6.9%)	
Mucinous	6 (3.5%)	4 (4.6%)	
Others	20 (11.6%)	6 (6.9%)	
Grading			
1	12 (7.0%)	8 (9.2%)	0.481
2	64 (37.2%)	30 (34.5%)	
3	80 (46.5%)	45 (51.7%)	
Not available	16 (9.3%)	4 (4.6%)	
Residual disease, n (%)			
No	19 (11.0%)	7 (8.0%)	0.739
Any residual	119 (69.2%)	63 (72.4%)	
Not available	34 (19.8%)	17 (19.5%)	
Chemotherapy regimen, n (%)			
Paclitaxel + carboplatin	128 (74.4%)	66 (75.9%)	0.776
Intraperitoneal	19 (11.0%)	9 (10.3%)	
Docetaxel + carboplatin	17 (9.9%)	10 (11.5%)	
Other	3 (1.7%)	0 (0%)	
Not available	5 (2.9%)	2 (2.3%)	
Cycles of total chemotherapy, median (range)	6 (1‐12)	6 (1‐9)	0.117

ASA, American Society of Anesthesiologists; CA‐125, cancer antigen 125; FIGO, International Federation of Gynecology and Obstetrics; HGSC, high‐grade serous carcinoma; PDS, primary debulking surgery.

**Figure 2:**
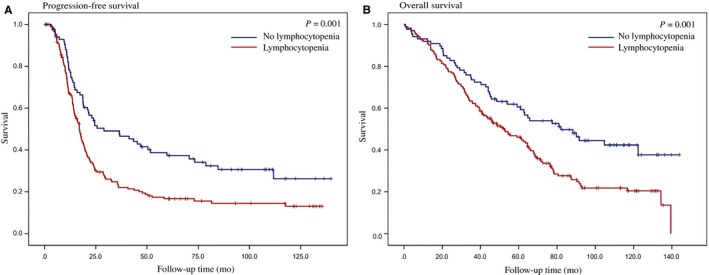
Kaplan‐Meier curves of progression‐free (A) survival and overall survival (B) stratified by lymphocytopenia in patients treated with PDS. PDS, primary debulking surgery

The median follow‐up duration was 54.3 months (range, 0.5‐144.0 months). At the time of analysis, 171 patients (66.0%) had died, and 191 (73.7%) had recurrences. The Kaplan‐Meier curves for OS and PFS are shown in Figure 2. Patients in the lymphocytopenia group had poorer PFS (*P *=* *0.001) and OS (*P *=* *0.001) than those in the nonlymphocytopenia group. The median PFS was 17.0 months (range 0.5‐135.5 months) in the lymphocytopenia group and 29.0 months (range 0.7‐139.8 months) in the nonlymphocytopenia group. The median OS was 52.6 months (range 0.5‐139.4 months) in the lymphocytopenia group and 82.0 months (range 0.7‐144.0 months) in the nonlymphocytopenia group.

The results of the multivariate analysis of PFS and OS in patients treated with PDS are shown in Table 4. The multivariate analysis showed that pretreatment lymphocytopenia was an independent prognostic factor associated with a higher risk of progression (HR, 1.73; 95% CI, 1.20‐2.49) and inferior OS (HR, 1.87; 95% CI, 1.27‐2.75). Under multivariate analysis in a continuous variable model, the absolute lymphocyte count retained significance as a prognostic factor for recurrence (HR 0.46; 95% CI, 0.32‐0.68) and OS (HR 0.42; 95% CI, 0.28‐0.63). The results of the univariate/multivariate analyses for all variables are in Tables S4 and S5.

**Table 4:** Multivariate analyses for progression‐free and overall survival using a Cox proportional hazards model with categorical variables and continuous variables in patients treated with PDS

Variables	PFS	OS
	HR (95% CI)	HR (95% CI)
Absolute lymphocyte count (Categorical variables^a^)		
≥1.49 × 10^9^/L	1	1
<1.49 × 10^9^/L	1.73 (1.20‐2.49)	1.87 (1.27‐2.75)
Absolute lymphocyte count (Continuous variables^b^)	0.46 (0.32‐0.68)	0.42 (0.28‐0.63)

ASA, American Society of Anesthesiologists; CI, confidence interval; FIGO, International Federation of Gynecology and Obstetrics; HR, hazard ratio; OS, overall survival; PDS, primary debulking surgery; PFS, progression‐free survival.

a The multivariate analysis was adjusted for categorical variables (age, ASA score, hemoglobin, absolute neutrophil count, CA‐125 level, FIGO stage, histology, residual disease after IDS, chemotherapy regimen, cycles of total chemotherapy).

b The multivariate analysis was adjusted for continuous variables (age, hemoglobin, absolute neutrophil count, CA‐125 level, cycles of total chemotherapy) and categorical variables (ASA score, FIGO stage, histology, residual disease after IDS, chemotherapy regimen).

## 4 DISCUSSION

In this study, we showed that pretreatment lymphocytopenia is an independent prognostic factor in advanced‐stage ovarian cancer patients treated with either NAC or PDS. Lymphocytopenia before initiation of NAC or PDS was found to independently correlate with PFS and OS. In addition, we showed that the absolute lymphocyte count was a significant adverse factor as a continuous variable in both the NAC and PDS cohorts, demonstrating that the magnitude of lymphocytopenia correlates with an inferior survival outcome.

Lymphocytopenia, which is frequently observed in ovarian cancer,^14^ may possibly reflect a state of tumor‐induced immune suppression. Although the actual mechanisms of tumor‐induced immune suppression are not fully known, the possibilities include that this suppression is the result of impaired homeostasis of lymphocytes and the consequence of chemokines and cytokines produced by the tumor microenvironment. Lymphocyte homeostasis requires the presence of dendritic cells and function of dendritic cells.^15^ The differentiation of dendritic cells is impaired by the overproduction of numerous cytokines and mediators such as interleukin‐6 and transforming growth factor β; this overproduction is caused by the tumor microenvironment.^16^ Lymphocytes of cancer also produce proapoptotic ligands such as Fas ligand, tumor necrosis factor, and programmed death‐1 ligand‐1 that produce inhibitory signals to suppress the cytotoxic activity of T cells.^17^ Changes in the cytokines provoke subtle modifications of the tumor microenvironment that interfere with the ability of the immune system to coordinate and efficiently control the tumor.

Recently, pretreatment lymphocytopenia has been suggested as an adverse prognostic factor in several solid tumors. Jang et al^18^ demonstrated that lymphocytopenia at diagnosis was a significant factor for poor survival outcomes in patients with primary central nervous system lymphoma. Joseph et al showed that pretreatment lymphocytopenia is an independent adverse prognostic factor in both muscle‐invasive and advanced bladder cancer. In addition, the study showed that the magnitude of lymphocytopenia correlates with inferior outcomes.^9^

Lymphocytopenia may reflect a state of immune suppression, which also diminishes the effect of chemotherapy.^10^ Ceze et al showed that the objective response rate was significantly lower in lymphocytopenia patients than in other colorectal cancer patients receiving chemotherapy (12.5% vs 40.2%; *P* = 0.004).^10^ There are several distinct mechanisms by which chemotherapy can modify the interactions between cancer cells and host immunity.^19^ Chemotherapy triggers cancer cell death, which restores or enhances the expression of cancer antigens and increases their susceptibility to attack from immune cells.^19^ Despite the many studies on tumor‐induced immune suppression as a prognostic factor, the association between tumor‐induced immune suppression and the status of the tumor microenvironment remains unclear. Tumor‐induced alterations in the differentiation of dendritic cells impair lymphocyte homeostasis and induce lymphocytopenia. Furthermore, dendritic cells attracted into the ovarian cancer microenvironment promote T cells to release large amounts of interleukin‐10, which prevents local T‐cell activation.^20^ Dendritic cells mediate immune suppression in the tumor microenvironment of ovarian cancer by suppressing T‐cell immunity through upregulation of B7‐H1^21^ and programmed cell death‐1.^22^ The immune response of the tumor microenvironment to ovarian cancer has a significant influence on clinical outcomes.^23^ Previous studies showed that the presence of CD+8 tumor‐infiltrating lymphocytes is associated with good survival outcomes.^24^, ^25^ However, Milne et al failed to find an association between absolute lymphocyte counts and CD8+ and CD20+ tumor‐infiltrating lymphocytes.^26^ They reported that absolute lymphocyte count seems to be not associated with the presence of tumor‐infiltrating lymphocytes.

Moreover, pretreatment lymphocytopenia can be a predictive biomarker to identify patients suitable for treatment to restore antitumor immunity in advanced‐stage ovarian cancer. In a retrospective analysis of over 167 adult patients treated with pembrolizumab or nivolumab for solid tumors, pretreatment and persistent lymphocytopenia during therapy were found to be correlated with unfavorable outcomes but also with less likelihood of immune‐related adverse events.^27^ Because blood‐based biomarkers have not been actively developed, further study is needed to determine whether less invasive and relatively easy blood‐based biomarkers can reveal the status of a tumor microenvironment.

If tumor‐associated immune suppression is a driver of cancer progression and of a poor response to chemotherapy, therapeutic strategies directed toward the restoration of antitumor immunity may improve the outcome. Administration of IL‐2 causes a transient increase in absolute lymphocyte counts, which has been associated with tumor response in metastatic melanoma.^28^ IL‐7 can also be used to increase lymphocytes (peripheral CD4+ and CD8+ T cells), resulting in immunomodulatory effects on T cells.^29^

This study has several limitations. The median follow‐up period was only 31.2 months in patients treated with NAC. Furthermore, this study design was a retrospective review of medical records, which depended on medical records or documentation and avoided potential recall bias. The strength of this study is that the optimal cutoff to define lymphocytopenia was calculated in the NAC cohort, and this optimal cutoff value was subsequently applied to the PDS cohort to demonstrate the independent prognostic effect of pretreatment lymphocytopenia.

In conclusion, pretreatment lymphocytopenia is a convenient and independent prognostic factor for advanced‐stage ovarian cancer patients. It may be associated with cancer‐induced immune suppression during tumor progression. Immuno‐oncologic agents restoring normal lymphocyte homeostasis may hold considerable promise in the treatment of advanced‐stage ovarian cancer.

## CONFLICT OF INTEREST

The authors declare that they have no conflicts of interest.
