# Supplementary figures and images for: Pretreatment lymphocytopenia is an adverse prognostic biomarker in advanced‐stage ovarian cancer
Source: Cancer Med. 2019 Jan 16;8(2):564–71. doi: 10.1002/cam4.1956 (PMC6382732; doi:10.1002/cam4.1956)

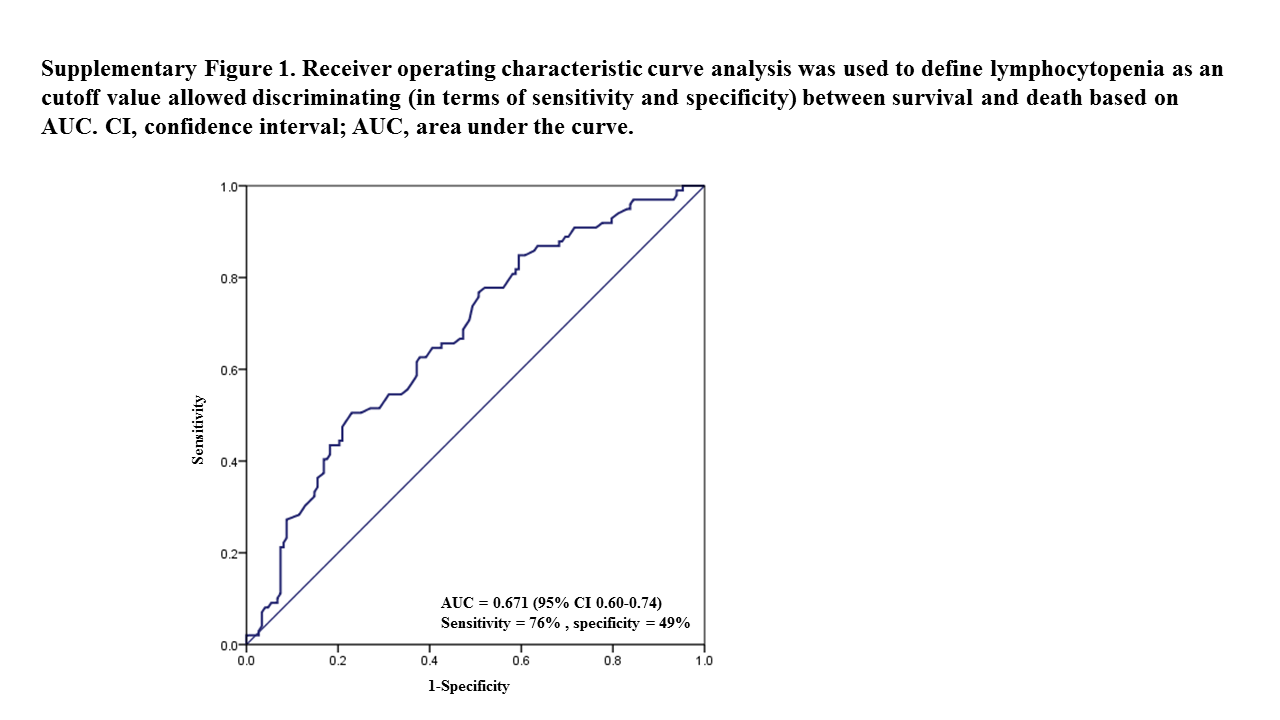

Supplement: Supplementary file 1 [file CAM4-8-564-s001.tif]
