# Supplementary material for: Pretreatment lymphocytopenia is an adverse prognostic biomarker in advanced‐stage ovarian cancer
Source: Cancer Med. 2019 Jan 16;8(2):564–71. doi: 10.1002/cam4.1956 (PMC6382732; doi:10.1002/cam4.1956)
Supplement: Supplementary file 2 [file CAM4-8-564-s002.docx]

Supporting Information Table S1. Absolute lymphocyte counts from 2006 to 2017, and P for trend according to years.

|  | NAC cohort | | PDS cohort | |
| --- | --- | --- | --- | --- |
| Years | Absolute lymphocyte count, median, cells/L | P for trend | Absolute lymphocyte count, median, cells/L | P for trend |
| 2006 | 1.00 × 10^9^/L | 0.323 | 1.38 × 10^9^/L | 0.051 |
| 2007 | 1.39 × 10^9^/L |  | 1.28 × 10^9^/L |  |
| 2008 | 1.69 × 10^9^/L |  | 1.60 × 10^9^/L |  |
| 2009 | 1.15 × 10^9^/L |  | 1.43 × 10^9^/L |  |
| 2010 | 1.25 × 10^9^/L |  | 1.21 × 10^9^/L |  |
| 2011 | 1.19 × 10^9^/L |  | 1.12 × 10^9^/L |  |
| 2012 | 1.22 × 10^9^/L |  | 1.10 × 10^9^/L |  |
| 2013 | 1.23 × 10^9^/L |  | 1.26 × 10^9^/L |  |
| 2014 | 1.25 × 10^9^/L |  | 1.06 × 10^9^/L |  |
| 2015 | 1.68 × 10^9^/L |  |  |  |
| 2016 | 1.46 × 10^9^/L |  |  |  |
| 2017 | 1.35 × 10^9^/L |  |  |  |

NAC, neoadjuvant chemotherapy; PDS, primary debulking surgery
