# Supplementary material for: Pretreatment lymphocytopenia is an adverse prognostic biomarker in advanced‐stage ovarian cancer
Source: Cancer Med. 2019 Jan 16;8(2):564–71. doi: 10.1002/cam4.1956 (PMC6382732; doi:10.1002/cam4.1956)
Supplement: Supplementary file 3 [file CAM4-8-564-s003.docx]

Supplementary Table 2. Univariate / Multivariate analyses for progression-free and overall survival using a Cox proportional hazards model with categorical variables in patients treated with NAC

a) Univariate analysis

| Variables | PFS | | OS | |
| --- | --- | --- | --- | --- |
|  | HR (95% CI) | P | HR (95% CI) | P |
| Age, years |  |  |  |  |
| ≤58 | 1 |  | 1 |  |
| >58 | 0.90 (0.67-1.21) | 0.491 | 1.33 (0.90-1.96) | 0.156 |
| ASA score |  |  |  |  |
| 1-2 | 1 |  | 1 |  |
| 3-4 | 1.16 (0.82-1.65) | 0.409 | 1.64 (1.03-2.61) | 0.036 |
| Hemoglobin level |  |  |  |  |
| ≥ 12.0 g/dL | 1 |  | 1 |  |
| < 12.0 g/dL | 1.40 (1.04-1.88) | 0.029 | 1.49 (1.00-2.21) | 0.049 |
| Absolute lymphocyte count |  |  |  |  |
| ≥ 1.49 × 10^9^/L | 1 |  | 1 |  |
| < 1.49 × 10^9^/L | 1.40 (1.03-1.90) | 0.031 | 1.89 (1.19-3.01) | 0.007 |
| Absolute neutrophil count |  |  |  |  |
| ≤ 7.5 × 10^9^/L | 1 |  | 1 |  |
| > 7.5 × 10^9^/L | 1.11 (0.75-1.63) | 0.606 | 1.24 (0.73-2.10) | 0.420 |
| CA-125 level |  |  |  |  |
| ≤ 1715.3 U/mL | 1 |  | 1 |  |
| > 1715.3 U/mL | 1.04 (0.77-1.41) | 0.783 | 1.12 (0.75-1.68) | 0.565 |
| FIGO stage |  |  |  |  |
| III | 1 |  | 1 |  |
| IV | 1.90 (1.40-2.59) | <0.001 | 1.45 (0.96-2.19) | 0.081 |
| Histology |  |  |  |  |
| HGSC | 1 |  | 1 |  |
| Non-HGSC | 1.35 (0.81-2.26) | 0.251 | 3.28 (1.88-5.72) | <0.001 |
| Residual disease |  |  |  |  |
| No | 1 |  | 1 |  |
| Any residual | 1.48 (1.1-2.00) | 0.010 | 1.56 (1.04-2.33) | 0.030 |
| Chemotherapy regimen |  |  |  |  |
| Paclitaxel + carboplatin | 1 |  | 1 |  |
| Others | 1.19 (0.84-1.69) | 0.336 | 0.93 (0.58-1.50) | 0.772 |
| Cycles of total chemotherapy |  |  |  |  |
| ≤8 | 1 |  | 1 |  |
| >8 | 1.19 (0.89-1.60) | 0.248 | 1.49 (1.00-2.21) | 0.050 |

b) Multivariate analysis

| Variables | PFS | | OS | |
| --- | --- | --- | --- | --- |
|  | HR (95% CI) | P | HR (95% CI) | P |
| Age, years |  |  |  |  |
| ≤58 | 1 |  | 1 |  |
| >58 | 0.91 (0.64-1.28) | 0.579 | 1.68 (1.04-2.73) | 0.034 |
| ASA score |  |  |  |  |
| 1-2 | 1 |  | 1 |  |
| 3-4 | 1.05 (0.69-1.58) | 0.831 | 1.85 (1.09-3.13) | 0.023 |
| Hemoglobin level |  |  |  |  |
| ≥ 12.0 g/dL | 1 |  | 1 |  |
| < 12.0 g/dL | 1.33 (0.95-1.87) | 0.099 | 1.35 (0.84-2.18) | 0.217 |
| Absolute lymphocyte count |  |  |  |  |
| ≥ 1.49 × 10^9^/L | 1 |  | 1 |  |
| < 1.49 × 10^9^/L | 1.50 (1.07-2.11) | 0.018 | 2.02 (1.21-3.40) | 0.008 |
| Absolute neutrophil count |  |  |  |  |
| ≤ 7.5 × 10^9^/L | 1 |  | 1 |  |
| > 7.5 × 10^9^/L | 1.20 (0.71-2.02) | 0.500 | 1.54 (0.68-3.49) | 0.298 |
| CA-125 level |  |  |  |  |
| ≤ 1715.3 U/mL | 1 |  | 1 |  |
| > 1715.3 U/mL | 1.08 (0.77-1.51) | 0.674 | 1.45 (0.92-2.30) | 0.114 |
| FIGO stage |  |  |  |  |
| III | 1 |  | 1 |  |
| IV | 2.07 (1.47-2.92) | <0.001 | 1.44 (0.86-2.36) | 0.142 |
| Histology |  |  |  |  |
| HGSC | 1 |  | 1 |  |
| Non-HGSC | 1.84 (1.03-3.28) | 0.040 | 4.45 (2.25-8.81) | <0.001 |
| Residual disease |  |  |  |  |
| No | 1 |  | 1 |  |
| Any residual | 1.37 (0.97-1.93) | 0.071 | 1.81 (1.08-3.02) | 0.024 |
| Chemotherapy regimen |  |  |  |  |
| Paclitaxel + carboplatin | 1 |  | 1 |  |
| Others | 0.93 (0.60-1.44) | 0.739 | 0.57 (0.30-1.10) | 0.096 |
| Cycles of total chemotherapy |  |  |  |  |
| ≤8 | 1 |  | 1 |  |
| >8 | 0.72 (0.51-1.03) | 0.071 | 0.59 (0.35-0.98) | 0.042 |

ASA, American Society of Anesthesiologists; CI, confidence interval; FIGO, International Federation of Gynecology and Obstetrics; HGSC, high-grade serous carcinoma; HR, hazard ratio; NAC, neoadjuvant chemotherapy; PFS, progression-free survival; OS, overall survival.
