# Supplementary material for: Pretreatment lymphocytopenia is an adverse prognostic biomarker in advanced‐stage ovarian cancer
Source: Cancer Med. 2019 Jan 16;8(2):564–71. doi: 10.1002/cam4.1956 (PMC6382732; doi:10.1002/cam4.1956)
Supplement: Supplementary file 4 [file CAM4-8-564-s004.docx]

Supplementary Table 3. Multivariate analyses for progression-free and overall survival using a Cox proportional hazards model with continuous variables in patients treated with NAC

| Variables | PFS | | OS | |
| --- | --- | --- | --- | --- |
|  | HR (95% CI) | P | HR (95% CI) | P |
| Age, years | 1.00 (0.99-1.01) | 0.730 | 1.04 (1.01-1.06) | 0.004 |
| ASA score |  |  |  |  |
| 1-2 | 1 |  | 1 |  |
| 3-4 | 1.08 (0.71-1.63) | 0.730 | 1.70 (0.97-2.97) | 0.064 |
| Hemoglobin level | 0.92 (0.82-1.03) | 0.146 | 0.94 (0.80-1.11) | 0.468 |
| Absolute lymphocyte count | 0.63 (0.46-0.88) | 0.006 | 0.31 (0.17-0.56) | <0.001 |
| Absolute neutrophil count | 1.03 (0.99-1.08) | 0.197 | 1.11 (1.04-1.18) | 0.002 |
| CA-125 level | 1.01 (0.98-1.06) | 0.151 | 1.05 (0.99-1.09) | 0.177 |
| FIGO stage |  |  |  |  |
| III | 1 |  | 1 |  |
| IV | 1.99 (1.42-2.81) | <0.001 | 1.44 (0.88-2.35) | 0.149 |
| Histology |  |  |  |  |
| HGSC | 1 |  | 1 |  |
| Non-HGSC | 2.02 (1.13-3.62) | 0.017 | 5.45 (2.75-10.79) | <0.001 |
| Residual disease |  |  |  |  |
| No | 1 |  | 1 |  |
| Any residual | 1.36 (0.97-1.93) | 0.077 | 2.05 (1.21-3.46) | 0.007 |
| Chemotherapy regimen |  |  |  |  |
| Paclitaxel + carboplatin | 1 |  | 1 |  |
| Others | 1.84 (1.04-3.25) | 0.706 | 0.59 (0.32-1.09) | 0.090 |
| Cycles of total chemotherapy | 0.94 (0.86-1.03) | 0.199 | 0.94 (0.83-1.07) | 0.368 |

ASA, American Society of Anesthesiologists; CI, confidence interval; FIGO, International Federation of Gynecology and Obstetrics; HGSC, high-grade serous carcinoma; HR, hazard ratio; NAC, neoadjuvant chemotherapy; PFS, progression-free survival; OS, overall survival.
