# Supplementary material for: Pretreatment lymphocytopenia is an adverse prognostic biomarker in advanced‐stage ovarian cancer
Source: Cancer Med. 2019 Jan 16;8(2):564–71. doi: 10.1002/cam4.1956 (PMC6382732; doi:10.1002/cam4.1956)
Supplement: Supplementary file 5 [file CAM4-8-564-s005.docx]

Supplementary Table 4. Univariate / Multivariate analyses for progression-free and overall survival using a Cox proportional hazards model with categorical variables in patients treated with PDS

a) Univariate analysis

| Variables | PFS | | OS | |
| --- | --- | --- | --- | --- |
|  | HR (95% CI) | P | HR (95% CI) | P |
| Age, years |  |  |  |  |
| ≤55 | 1 |  | 1 |  |
| >55 | 0.90 (0.69-1.18) | 0.461 | 1.37 (1.03-1.83) | 0.033 |
| ASA score |  |  |  |  |
| 1-2 | 1 |  | 1 |  |
| 3-4 | 2.18 (1.36-3.51) | <0.001 | 2.32 (1.40-3.83) | 0.001 |
| Hemoglobin level |  |  |  |  |
| ≥ 12.0 g/dL | 1 |  | 1 |  |
| < 12.0 g/dL | 1.30 (0.98-1.73) | 0.067 | 1.27 (0.94-1.72) | 0.115 |
| Absolute lymphocyte count |  |  |  |  |
| ≥ 1.49 × 10^9^/L | 1 |  | 1 |  |
| < 1.49 × 10^9^/L | 1.73 (1.26-2.37) | 0.001 | 1.72 (1.23-2.41) | 0.002 |
| Absolute neutrophil count |  |  |  |  |
| ≤ 7.5 × 10^9^/L | 1 |  | 1 |  |
| > 7.5 × 10^9^/L | 1.14 (0.83-1.55) | 0.417 | 1.22 (0.88-1.70) | 0.232 |
| CA-125 level |  |  |  |  |
| ≤ 1791.7 U/mL | 1 |  | 1 |  |
| > 1791.7 U/mL | 0.95 (0.65-1.38) | 0.767 | 0.99 (0.67-1.47) | 0.967 |
| FIGO stage |  |  |  |  |
| III | 1 |  | 1 |  |
| IV | 1.49 (1.12-1.99) | 0.006 | 1.21 (0.88-1.67) | 0.234 |
| Histology |  |  |  |  |
| HGSC | 1 |  | 1 |  |
| Non-HGSC | 0.92 (0.66-1.29) | 0.630 | 1.16 (0.82-1.65) | 0.412 |
| Residual disease |  |  |  |  |
| No | 1 |  | 1 |  |
| Any residual | 1.57 (0.97-2.53) | 0.064 | 2.72 (1.38-5.34) | 0.004 |
| Chemotherapy regimen |  |  |  |  |
| Paclitaxel + carboplatin | 1 |  | 1 |  |
| Others | 0.83 (0.60-1.15) | 0.268 | 0.87 (0.61-1.24) | 0.449 |
| Cycles of total chemotherapy |  |  |  |  |
| ≤6 | 1 |  | 1 |  |
| >6 | 1.05 (0.75-1.48) | 0.768 | 1.12 (0.78-1.61) | 0.529 |

b) Multivariate analysis

| Variables | PFS | | OS | |
| --- | --- | --- | --- | --- |
|  | HR (95% CI) | P | HR (95% CI) | P |
| Age, years |  |  |  |  |
| ≤55 | 1 |  | 1 |  |
| >55 | 0.69 (0.49-0.96) | 0.029 | 1.26 (0.886-1.80) | 0.197 |
| ASA score |  |  |  |  |
| 1-2 | 1 |  | 1 |  |
| 3-4 | 3.10 (1.76-5.46) | <0.001 | 2.49 (1.38-4.51) | 0.003 |
| Hemoglobin level |  |  |  |  |
| ≥ 12.0 g/dL | 1 |  | 1 |  |
| < 12.0 g/dL | 1.37 (0.97-1.93) | 0.073 | 1.22 (0.85-1.76) | 0.284 |
| Absolute lymphocyte count |  |  |  |  |
| ≥ 1.49 × 10^9^/L | 1 |  | 1 |  |
| < 1.49 × 10^9^/L | 1.73 (1.20-2.49) | 0.003 | 1.87 (1.27-2.75) | 0.002 |
| Absolute neutrophil count |  |  |  |  |
| ≤ 7.5 × 10^9^/L | 1 |  | 1 |  |
| > 7.5 × 10^9^/L | 1.04 (0.71-1.52) | 0.839 | 1.07 (0.71-1.61) | 0.751 |
| CA-125 level |  |  |  |  |
| ≤ 1791.7 U/mL | 1 |  | 1 |  |
| > 1791.7 U/mL | 0.95 (0.65-1.38) | 0.767 | 0.99 (0.67-1.47) | 0.967 |
| FIGO stage |  |  |  |  |
| III | 1 |  | 1 |  |
| IV | 1.55 (1.07-2.25) | 0.022 | 1.20 (0.81-1.78) | 0.375 |
| Histology |  |  |  |  |
| HGSC | 1 |  | 1 |  |
| Non-HGSC | 0.57 (0.35-0.92) | 0.022 | 0.69 (0.40-1.19) | 0.177 |
| Residual disease |  |  |  |  |
| No | 1 |  | 1 |  |
| Any residual | 1.91 (1.12-3.28) | 0.018 | 2.71 (1.30-5.66) | 0.008 |
| Chemotherapy regimen |  |  |  |  |
| Paclitaxel + carboplatin | 1 |  | 1 |  |
| Others | 0.74 (0.51-1.10) | 0.132 | 0.80 (0.53-1.21) | 0.375 |
| Cycles of total chemotherapy |  |  |  |  |
| ≤6 | 1 |  | 1 |  |
| >6 | 1.00 (0.65-1.54) | 0.988 | 1.47 (0.95-2.27) | 0.084 |

ASA, American Society of Anesthesiologists; CI, confidence interval; FIGO, International Federation of Gynecology and Obstetrics; HGSC, high-grade serous carcinoma; HR, hazard ratio; PDS, primary debulking surgery; PFS, progression-free survival; OS, overall survival.
