# Supplementary material for: Pretreatment lymphocytopenia is an adverse prognostic biomarker in advanced‐stage ovarian cancer
Source: Cancer Med. 2019 Jan 16;8(2):564–71. doi: 10.1002/cam4.1956 (PMC6382732; doi:10.1002/cam4.1956)
Supplement: Supplementary file 6 [file CAM4-8-564-s006.docx]

Supplementary Table 5. Multivariate analyses for progression-free and overall survival using a Cox proportional hazards model with continuous variables in patients treated with PDS

| Variables | PFS | | OS | |
| --- | --- | --- | --- | --- |
|  | HR (95% CI) | P | HR (95% CI) | P |
| Age, years | 0.99 (0.98-1.00) | 0.367 | 1.02 (0.99-1.03) | 0.068 |
| ASA score |  |  |  |  |
| 1-2 | 1 |  | 1 |  |
| 3-4 | 2.55 (1.44-4.51) | 0.001 | 2.93 (1.41-6.11) | 0.004 |
| Hemoglobin level | 0.88 (0.77-1.05) | 0.082 | 0.91 (0.80-1.04) | 0.163 |
| Absolute lymphocyte count | 0.46 (0.32-0.68) | <0.001 | 0.42 (0.28-0.63) | <0.001 |
| Absolute neutrophil count | 0.99 (0.93-1.05) | 0.696 | 0.97 (0.91-1.04) | 0.402 |
| CA-125 level | 1.02 (0.98-1.06) | 0.512 | 1.05 (0.99-1.08) | 0.345 |
| FIGO stage |  |  |  |  |
| III | 1 |  | 1 |  |
| IV | 1.56 (1.08-2.25) | 0.018 | 1.20 (0.81-1.78) | 0.358 |
| Histology |  |  |  |  |
| HGSC | 1 |  | 1 |  |
| Non-HGSC | 0.60 (0.37-0.99) | 0.049 | 0.98 (0.57-1.68) | 0.938 |
| Residual disease |  |  |  |  |
| No | 1 |  | 1 |  |
| Any residual | 1.87 (1.09-3.23) | 0.024 | 2.93 (1.41-6.11) | 0.004 |
| Chemotherapy regimen |  |  |  |  |
| Paclitaxel + carboplatin | 1 |  | 1 |  |
| Others | 0.74 (0.51-1.09) | 0.129 | 0.86 (0.57.-1.30) | 0.474 |
| Cycles of total chemotherapy | 1.06 (0.94-1.20) | 0.341 | 1.02 (0.88-1.18) | 0.825 |

ASA, American Society of Anesthesiologists; CI, confidence interval; FIGO, International Federation of Gynecology and Obstetrics; HGSC, high-grade serous carcinoma; HR, hazard ratio; PDS, primary debulking surgery; PFS, progression-free survival; OS, overall survival.
